# Assessment of the Potential Impacts of Wheat Plant Traits across Environments by Combining Crop Modeling and Global Sensitivity Analysis

**DOI:** 10.1371/journal.pone.0146385

**Published:** 2016-01-22

**Authors:** Pierre Casadebaig, Bangyou Zheng, Scott Chapman, Neil Huth, Robert Faivre, Karine Chenu

**Affiliations:** 1 INRA, UMR1248 AGIR, 31326 Castanet-Tolosan, France; 2 CSIRO Agriculture, Queensland Bioscience Precinct, 306 Carmody Road, St. Lucia, QLD 4067, Australia; 3 Queensland Alliance for Agriculture and Food Innovation (QAAFI), The University of Queensland, St Lucia, QLD 4350, Australia; 4 CSIRO Agriculture, 203 Tor Street, Toowoomba, QLD 4350, Australia; 5 INRA, UR875 MIAT, 31326 Castanet-Tolosan, France; 6 Queensland Alliance for Agriculture and Food Innovation (QAAFI), The University of Queensland, 203 Tor Street, Toowoomba, QLD 4350, Australia; Pennsylvania State University, UNITED STATES

## Abstract

A crop can be viewed as a complex system with outputs (e.g. yield) that are affected by inputs of genetic, physiology, pedo-climatic and management information. Application of numerical methods for model exploration assist in evaluating the major most influential inputs, providing the simulation model is a credible description of the biological system. A sensitivity analysis was used to assess the simulated impact on yield of a suite of traits involved in major processes of crop growth and development, and to evaluate how the simulated value of such traits varies across environments and in relation to other traits (which can be interpreted as a virtual change in genetic background). The study focused on wheat in Australia, with an emphasis on adaptation to low rainfall conditions. A large set of traits (90) was evaluated in a wide target population of environments (4 sites × 125 years), management practices (3 sowing dates × 3 nitrogen fertilization levels) and *CO*_2_ (2 levels). The Morris sensitivity analysis method was used to sample the parameter space and reduce computational requirements, while maintaining a realistic representation of the targeted trait × environment × management landscape (∼ 82 million individual simulations in total). The patterns of parameter × environment × management interactions were investigated for the most influential parameters, considering a potential genetic range of +/- 20% compared to a reference cultivar. Main (i.e. linear) and interaction (i.e. non-linear and interaction) sensitivity indices calculated for most of APSIM-Wheat parameters allowed the identification of 42 parameters substantially impacting yield in most target environments. Among these, a subset of parameters related to phenology, resource acquisition, resource use efficiency and biomass allocation were identified as potential candidates for crop (and model) improvement.

## Introduction

Progress in plant breeding is limited by the ability to predict plant phenotype based on its genotype, especially for complex traits such as yield. Suitably constructed process-based models provide a mean to reduce this gap in particular by dissecting the complexity of the genotype-environment interactions and by simulating expected impacts in various environmental conditions [1–3], including consideration of future climates [4, 5].

From a modeling point of view, crops are complex systems arising from interactions among genetic determinants, physiological processes, pedo-climatic factors and management practices. The combination of these elements, which are either chosen (cultivar and management) or given (soil and climate) in any sown crop, generates greatly variable stress patterns [6, 7] and results in high genotype (G) × environment (E) × management (M) interactions. A number of such interactions has been reported in the literature [8, 9], and sources of yield variation, especially in rainfed systems, commonly arise primarily from the genotype × environment (G×E) interactions, rather than the genotype (G), i.e. G×E > G as observed for field pea in Canada [10], sunflower in Argentina [11], sorghum in Australia [12], wheat in north-east Australia [13] and globally [14] and maize in Midwestern US states [15, 16] Modeling approaches have been developed to better understand G×E×M interactions and attempt to take advantage of genetic and environmental resources more efficiently. For example, Hammer et al. [17] show that the multi-year risk of crop failure for farms within a given sorghum region can be reduced by the adoption of better combinations of GxM (“local G” and “local M”) compared to use of the combination of “global G” and “global M” that would be adopted if using the entire sorghum production area.

Process-based crop models are useful tools to integrate scientific knowledge and simulate varietal or management impacts on productivity in the target population of environments (TPE), i.e. the set of environments to which newly bred varieties need to be adapted [18, 19]. Hence, the predictive capability of crop models is used to explore the complex G×E×M landscape and assists breeding programs to take advantage of genetic and environmental resources more efficiently [2, 20, 21]. While such models are based on mathematical equations translating biological processes in relation to crop growth and development, their parameters can be controlled to mimic effects of genotypic variability and explore the G×E×M landscape using *virtual genotypes* [22, 23]. Numerical exploration of crop models for the target population of environments thus allows exploration of the entire G×E×M landscape, assuming that the crop simulation model gives a credible description of the biological system.

To be relevant, exploration of the G×E×M landscape has to be applied to environments and management practices related to targeted production systems. A recent study characterized the drought environment of rainfed wheat for the Australian target population of environments [7], an interesting target given that Australia is the fourth wheat exporter worldwide and that Australian wheat crops have to adapt to a high variability (spatial and inter-annual) in drought patterns, which strongly impedes crop breeding [9, 13, 24] The Australian wheatbelt extends ca. 13 million ha (Australian Bureau of Statistics, 2013) and has soils ranging from shallow sandy to deep clay soils and include temperate, Mediterranean and subtropical climates [25, 26]. Chenu et al. [7] undertook a simulation-based study (60 sites × 5 initial soil moisture × 5 sowing dates) to capture the variability in environmental and management conditions of this TPE. To study genotypic variation in such a TPE raises computational challenges if variations in multiple plant traits with high granularity (resolution) are desired, i.e. requiring the simulation of many levels of small increment for each of the factors explored.

The APSIM (www.apsim.info) Wheat model [27–29] is used to simulate crop performance as a function of plant traits, pedo-climatic variability and management practices. This model has been extensively used and tested across Australia [6, 27, 28, 30]. Numerical experiments with crop models allow exploration of large G×E×M landscape. However, sampling the G×E×M landscape using a factorial design with as few as six levels for each parameter of the APSIM-Wheat model in the Australian TPE considered in this study would require to perform 9.72 × 10^73^ simulations. Such an approach would require absurdly high computing resource and could be considered as partly wasteful given that it considers all parameters including those of minimal importance. An alternative is to apply a numerical method designed to more efficiently explore complex landscapes. For instance, global sensitivity analysis allows investigation of how the uncertainty in the output of a model can be apportioned to different sources of uncertainty in the model input [31, 32].

Few computational studies have used sensitivity analysis to address cropping problems, e.g assessing the impact of phenology and management on sugarcane yield in various environments [33], the influence of geometrical and topological traits on light interception efficiency of apple trees [34] and the impact of physiological traits on wheat grain yield and protein concentration in Europe [35]. Recently, Zhao at al. [36] performed a sensitivity analysis on the APSIM-Wheat model with a focus on a narrow set of cultivar-specific traits (10 parameters) with the aim to improve an incoming calibration step.

**Fig 1:**
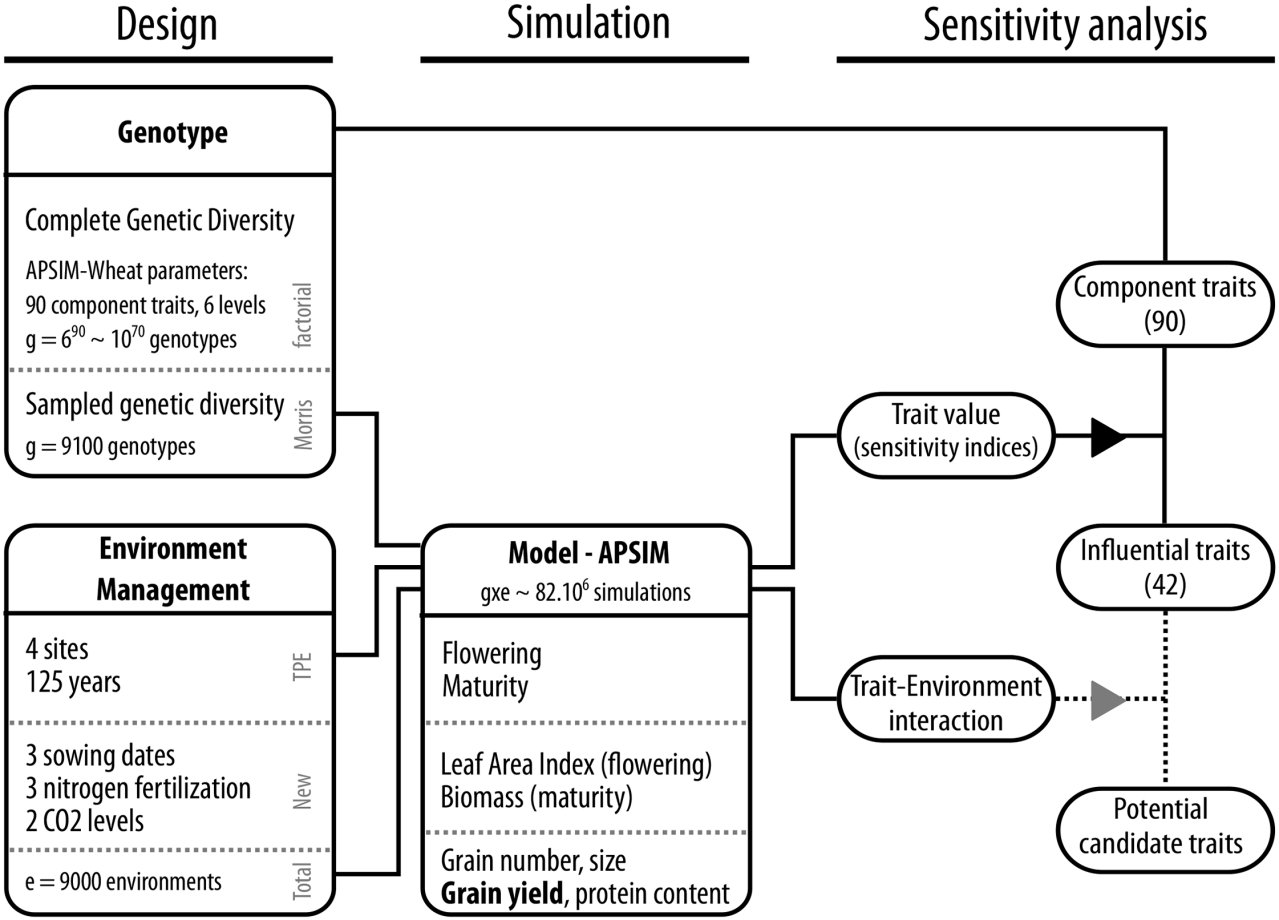
Framework of crop model simulation and the sensitivity approach used to assess the potential impact of plant traits. A global sensitivity analysis was applied on the APSIM-Wheat crop model to identify potential candidate traits for yield improvement in a large population of environments. This workflow presents how the “genetic diversity” was considered, sampled and screened *in silico*. In summary, 90 independent APSIM-Wheat parameters considered as “component traits”, associated with the main physiological processes that are modeled, were selected to reflect a potential genetic variability. Each of the 90 component traits was assumed to vary in a ± 20% range around the value for the reference cultivar *Hartog* and the Morris method [37, 38] was used to sample the total parameter space (90 traits, 6 levels, 100 reps; i.e. 9100 “genotypes”). Simulations for those genotypes were performed with APSIM-Wheat (Version 7.5). The impact of the 90 component traits were considered for 8 output variables (“integrated traits”, which result from the complexity of the dynamic modeling of development and growth). The impact on crop yield allowed to screen component traits for influential traits (n = 42) in the target population of environments while a study on trait × environment interactions was used to explore their variability across environments.

The aims of this paper were (i) to assess the impact of a suite of physiological traits on yield for Australian rain-fed wheat crops and (ii) to evaluate how the value of such traits varies across environments and in relation to other traits. A large set of traits (103) were evaluated in APSIM-Wheat for a wide population of environments related to four representative locations [7, 24] and 125 years of historical records of weather data (Fig 1). In addition to this representative set of 500 environment conditions, simulations were performed for three sowing dates, three levels of nitrogen fertilization and two levels of *CO*_2_ (i.e. 9000 conditions in total) to assess the effects of management and *CO*_2_ factors. We used a global sensitivity analysis to determine the effects of all traits on yield for all the conditions studied (i.e. each site × year × management combination). Traits found to have substantial and frequent impacts on yield were further studied through variance analysis to investigate the influence of environmental-factors and their impact on integrated traits such as plant leaf area, biomass production, and grain size and number.

## Material and methods

### Overview

A global sensitivity analysis was applied on the APSIM-Wheat crop model to identify potential candidate traits for yield improvement in a large population of environments. Fig 1 describes this workflow, showing how the “genetic diversity” was considered, sampled and screened *in silico*. In summary, from 516 parameters of the APSIM-Wheat model (with a broad-sense definition of parameter which included e.g. physical constants, optional parameters used for other crops and parameters repeated for different stages), 90 independent parameters that could be considered as “component traits” were selected to reflect a potential genetic variability. Each of the 90 component traits was assumed to vary in a ± 20% range around the value for the reference cultivar *Hartog*. The number of considered traits prevented the use of a factorial design, and so the Morris method [37, 38] was used to sample the total parameter space (90 traits, 6 levels, 100 reps; i.e. 9100 “genotypes”). Simulations for those genotypes were performed with APSIM-Wheat (Version 7.5) for (1) 4 locations and 125 years (from 1889 to 2013) to test the impact of component traits in the TPE (Table 1) and (2) for 3 sowing dates (i.e. early, TPE-level and late), 3 levels of nitrogen (i.e. low, TPE-level and high fertilization) and 2 levels of *CO*_2_ (380 and 555 ppm to represent *CO*_2_ level in 2010 and 2050) to test trait impact in other environmental conditions related to farmer management practices and future climates. The impact of the 90 component traits were considered for 8 output variables (“integrated traits”, Table 2) related to phenology (flowering and maturity dates), leaf development (Leaf Area Index at flowering), biomass production (at maturity), and grains (grain number, size, protein and yield). Overall 42 component traits were identified as “influential” (i.e. main average impact on yield greater than 20 kg ha-1) and considered as potential candidates to improve yield in the TPE. They were analyzed in more detail with a variance analysis. Several interesting traits related to phenology, resource acquisition, resource use efficiency and biomass allocation were studied in more detail as their impact could be related to specific environmental factors. A more complete description of the workflow and analysis is given below.

**Table 1:** Characteristics of the locations, soils and management representing the target population of environments. Plant available water capacity (PAWC) is indicated for each soil, as well at the level of initial soil water used in the simulations (median of plant available water at sowing which was estimated from [7]). Applied nitrogen dose are indicated by “a/b/c”: respectively, the fertilization applied at sowing (*a*), at the stage “end of tillering” (*b*) and at the stage “mid-stem elongation” (*c*). Annual and seasonal (1-May to 1-Nov) climatic data were considered for 1889-2013.

	Emerald	Narrabri	Yanco	Merredin
latitude (degree)	-23.53	-30.32	-34.61	-31.5
longitude (degree)	148.16	149.78	146.42	118.22
rainfall pattern	summer dominant	summer dominant	evenly distributed	winter dominant
annual rainfall (mm)	635	650	425	303
seasonal rainfall (mm)	170	249	228	209
seasonal PET (mm)	843	640.2	462.2	601.6
daily mean temperature (celcius)	18.4	13.9	11.9	13.1
daily mean radiation (MJ.m^-2^)	18.3	15.7	13.3	14.5
soil type	black vertosol	grey vertosol	brown sodosol	shallow loamy duplex
PAWC (mm)	133.5	217.5	190.8	101.1
sowing date	15/05	15/05	15/05	15/05
sowing PAWC (mm)	132	175	99	39
initial nitrogen (kg.ha^-1^)	30	30	50	30
applied nitrogen (kg.ha^-1^)	50/0/0	130/0/0	40/40/40	20/20/30

**Table 2:** Description of integrated traits (APSIM-Wheat output variables) and environmental indices included in the analysis. Environment indices were computed for the sowing-harvest period, for all considered environments. Water-deficit index correspond to the simulated water supply-demand ratio and relates to the degree to which the water available to the roots matches the plant water demand [7]. Nitrogen stress index relates to the level of nitrogen stress on photosynthesis. Stress indices are expressed as scalars so that values range from 0 (low stress) to 1 (high stress).

Type	Variable	Description	Unit
Crop	Flowering	Flowering date	day
Crop	Maturity	Maturity date	day
Crop	LAI	Leaf area index at flowering	-
Crop	Grain Size	Dry biomass of an individual grain	g
Crop	Grain Number	Grain number	grain
Crop	Grain Protein	Grain protein content	%
Crop	Biomass	Crop aerial dry biomass at harvest	t ha^-1^
Crop	Yield	Crop grain yield at harvest	t ha^-1^
Environment	Water	Average soil water deficit ratio	-
Environment	Nitrogen	Average nitrogen stress factor	-

### Simulations and sensitivity analysis

**Fig 2:**
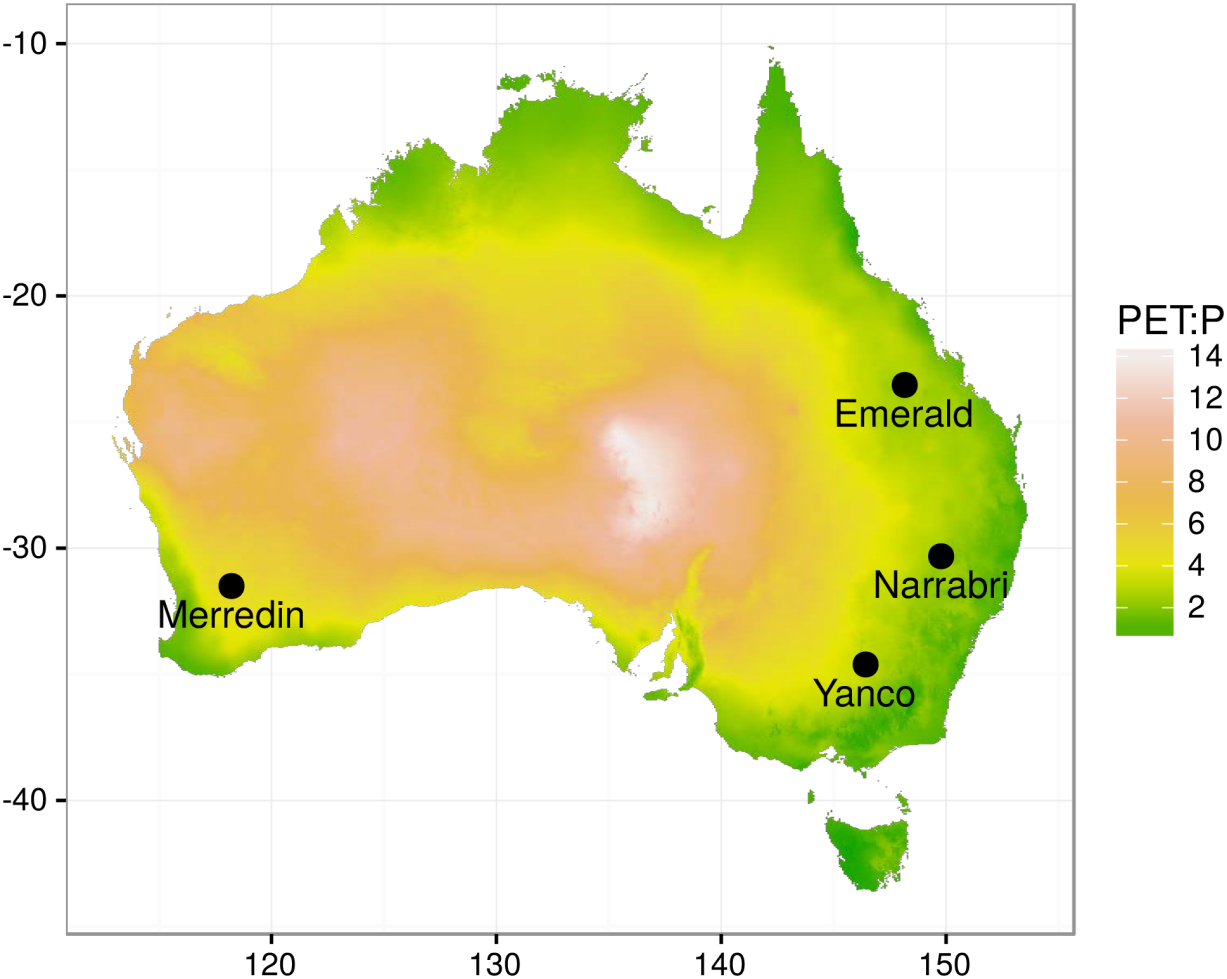
Map of the studied sites and climatic variability in aridity index. The map shows potential evapotranspiration over precipitation ratio (1 / aridity index, data from Zomer et al. [41]), points correspond to locations sampled in the target population of environments.

A global sensitivity analysis was performed on parameters of the crop model APSIM-Wheat version 7.5 [28, 29] to assess their impact on yield in the Australian wheatbelt (Figs 1–2). Five main steps were followed: (1) listing the input APSIM-Wheat parameters (input factors) to be included in the analysis, (2) setting the variation range for each factor, (3) sampling the parameter space with the Morris method, (4) simulating the virtual experiment with APSIM-Wheat and (5) computing the sensitivity indices to assess the impact of each factor singly (main effect) or in combination (interaction).

#### 1. Defining input factors

As for most crop models, APSIM-Wheat has parameters (S1 Table) that specify quantitative effect of processes related directly or indirectly to crop growth and development [27–29, 39]. Those parameters are typically either single values or arrays of paired vectors (S1 Table and S1 Fig), in which case one vector relates to the piloting a state variable (x; e.g. stage values) and the second one corresponds to the considered trait (y; e.g. values of root biomass partitioning for the different key stages considered). Each defined value, whether it is a single-value parameter or a point in an array can be considered as a parameter; in which case, APSIM-Wheat has 516 parameters (v. 7.5, as documented in Zheng et al. [29]). As all numerical coefficients in APSIM are completely external to the code, these “parameters” actually included a lot of constants and coefficients that would never be changed. Not all parameters were considered when assessing the impact of plant traits on crop performance as (1) four parameters representing soil physics and general physical constants (e.g. ammonium diffusion rate) were not considered, (2) 22 parameters deliberately set to have no impact on wheat crops (e.g. multiplicative scalars which are set to 1.0 by default in the released version of APSIM-Wheat) were not considered and (3) values in vectors (parameter arrays) were considered as dependent parameters, counting one parameter for the whole “function”.

This process greatly reduced the number of parameters to 103 (62 single values and 41 functions), yet there was no information loss on the system description. In addition, some parameters were grouped [38] to avoid aberrant situations and computational errors (e.g new min thresholds being greater than new max thresholds). In total, 20 parameters (annotated with * in S1 Table) were grouped into 7 “meta-parameters” that govern their variation (e.g. nitrogen demand, leaf expansion processes). Overall, 90 parameters (*p* = 103 − 20 + 7 = 90) were considered in the sensitivity analysis, with all the crop processes from APSIM-Wheat being tested (i.e. no process was removed from the analysis).

#### 2. Setting the variation range

The range of parameter values is biologically constrained by the genetic diversity existing in wheat. However, most crop models have typically been designed to only simulate major differences among cultivars (e.g. phenology), as their primary aim has been to address crop management problems. As a result, crop models such as APSIM-Wheat only have a few parameters that are by default considered as cultivar-dependent, while all the other parameters are assumed to be constant for the species. Given the lack of knowledge related to the range of the genetic variability existing for most of the model parameters, a fixed range of 40% variation for all parameters was tested in the sensibility analysis. Where possible, equal variation around the nominal value (± 20%) was considered, but for hard-bounded parameters (e.g scalars comprised between 0 and 1) the 40% variation was considered below (or above) the nominal value. Nominal values were considered for the reference cultivar *Hartog* and scaled using two consecutive rules: (1) direct scaling of the single value, or of all the *y* vector for function parameters (e.g. proportion of biomass partitioned to the roots at different stages) and (2) scaling only one single point in the *x* or *y* vector when this improved the biological meaning (e.g. threshold of leaf-expansion sensitivity to water deficit). S1 Fig illustrates the shape and variation range for function parameters studied in this sensitivity analysis.

#### 3. Sampling the parameter space

We used the Morris method [37] as implemented by Campagnolo et al. [38] to sample the parameter space and compute sensitivity indices. The method consists in a discretization of the input space for each factor (n = 6 levels), then performing a given number of one-at-a-time (OAT) design (r = 100) on the 90 parameters. The OAT designs were randomly chosen in the input space, and the variation direction was also random but their dispersion in the input space was maximized [38]. The repetition 100 times of these steps allowed the estimation of elementary effects for each input factor [37]. The implementation in the *sensitivity* R package used the space-filling optimization of the design [38]. Parameter design was normalized to account for the different magnitudes in input factors (parameters expressed in different units).

Considering the total number of input factors and the sampling conditions, the total size of parameter design was 90 + 1 × 100 = 9100, where each sample (i.e. set of parameter values) can be interpreted as a virtual genotype (i.e. 9100 in total). The numerical sampling of the parameter space can be viewed as an exploration of virtual genotype materials where there is no restriction in the combination of traits considered (i.e. no genetic linkage or epistasis).

#### 4. Design of experiments for crop simulations

The previous parameter design (i.e. the 9100 parameter combinations) was used with the APSIM-Wheat crop model to simulate 9100 virtual genotypes. APSIM-Wheat simulations were first done for the target population of environments (TPE, i.e. control conditions, Table 1) defined by 4 sites (Emerald, Narrabri, Yanco and Merredin; Fig 2, Table 1) and 125 years (1889-2013) of climatic data (4 × 125 = 500 environments). Crop management in these simulations (Table 1) was chosen to mimic local farming practices [7]. Additional simulations were performed for 3 sowing dates 21/04; 15/05; 07/06), 3 nitrogen fertilization levels (low: 50% of TPE-level, TPE-level and high fertilization: TPE-level plus 50 kg.ha^-1^) and 2 *CO*_2_ levels (TPE-level of 380 ppm and 555 ppm to represent *CO*_2_ level in 2010 and 2050) to explore the impact of parameters in contrasting N and *CO*_2_ conditions.

Nitrogen fertilization rules followed an APSIM decision model: at sowing, nitrogen was applied as nitrate in Merredin and as urea in the rest of the wheatbelt. In Yanco, fertilisation at “end of tillering” stage only occurred if cumulative rainfall since sowing was greater than 100 mm, and fertilisation at “mid-stem elongation” stage only occurred if plant available water was greater than 60% of the PAWC. At Merredin, fertilisation at “mid-stem elongation” only occurred if plant available water was greater than 60 mm.

Overall, 9000 (3 × 3 × 2 × 500 = 9000) environmental conditions were tested, and 81.9 million of crops (9100 × 9000) were simulated on the CSIRO distributed computing cluster which can sustain a peak throughput of approximately 8000 simultaneous processes [40]. Parameter impacts were tested on eight output variables from APSIM (Table 2): number of days from sowing to flowering and from sowing to maturity, leaf area index (LAI), biomass production, the number, size and protein content of grains and yield.

The baseline simulations were performed with the reference cultivar Hartog to estimate environmental indices (Table 2) and crop performance in each environment. In addition, the growing environments were characterized in terms of drought environment types, as described in Chenu et al. [7].

#### 5. Computation of sensitivity indices

Sensitivity indices were computed as statistics of elementary effect, i.e effect of the factor for each repetition [37, 38]. In this approach, the main effect (noted $\mu_{i}^{*}$ in Iooss et al. [42]) is a measure of the influence of the *i*-th input on the output, and is calculated as the mean of the absolute value of the elementary effects. The larger $\mu_{i}^{*}$ is, the more the input contributes to the dispersion of the output. The interaction effect (*σ*_*i*_ in Iooss et al. [42]), is a measure of non-linear and/or interaction effects of the *i*-th input. *σ*_*i*_ is computed as the standard deviation of the elementary effects. An input with a large *σ*_*i*_ can be considered as having non-linear effects or being involved in an interaction with at least another input. We also computed a standardized sensitivity index to be able to compare indices across different output variables and growing conditions. In this case, for each growing environment, the model output variables were standardized ($x^{\prime }=\frac{x-mean(x)}{sd(x)}$) before computing elementary effects and sensitivity indices.

### Clustering parameters according to their impact

All of the considered parameters were subdivided into three groups according to the mean value of their main effect in the target population of environments (i.e. mean of $\mu_{i}^{*}$ across environments): (1) *null impact* group, in which parameters had no impact on crop yield in any environments (2) *low impact* group, in which the parameters had an average $\mu_{i}^{*}$ lower or equal to 0.02 t ha^-1^ and (3) *impactful* group, in which parameters had an average main effect on yield that was greater than 0.02 t ha^-1^. A hierarchical clustering based on Ward distance was applied to the matrix of *impactful* parameters and the eight output variables (averaged across environments) to group these parameters and identify those with similar patterns of effect on output variables.

### Analysis of trait × environment interactions and computation of environment indices

For plant traits corresponding to influential parameters, we conducted a variance analysis to assess the effects of environmental factors on the variability of impact. Hence, for each trait, a linear model (Eq 1.) was fitted with environment-related factors considered as categorical fixed effects and with no interaction as *Y* = *α*_*site*_ + *β*_*sowing*_ + *γ*_*CO*_2__ + *δ*_*nitrogen*_ + *ϵ* with *Y*, vector of main effects for one trait in the 9000 environments; *α*, *β*, *γ*, *δ* are the additive main effects of the levels in factor *CO*_2_, site, sowing and nitrogen, respectively (Eq 1.). The effect of each environmental factor (*e*) on trait impact was estimated by the proportion of total sum of square (*η*^2^) as *SS*_*e*_/(*TSS*). Note that both the effect of “uncontrollable” environmental factors (i.e. climate) and the interactions among factors were pooled in the residuals.

Finally, we considered the response to the environment of a small subset of candidate traits and defined several environmental stress indices (Table 2) to further illustrate the ecophysiological basis of trait × environment interactions. Using the ASPIM Wheat model, daily computed indices related to water and nitrogen stresses were averaged for the duration of the crop cycle. In the model, water-stress is computed as a function of the soil water extractable by roots (water supply) and potential crop transpiration (water demand) [7]. The nitrogen-stress determined the limiting nitrogen level affecting leaf photosynthesis [29]. In this study, both indices were set to range from 0 (no-stress) to 1 (extreme stress) to allow comparison between stress indices.

### Software

All data processing, statistical analysis and graphics were performed with R 3.1.0 [43] with additional R packages *dplyr* (data processing [44]), *sensitivity* (sensitivity analysis, version 1.10.1 [45]) and *ggplot2* (visualization [46]).

## Results

### A target population of environments with contrasting environmental conditions

**Fig 3:**
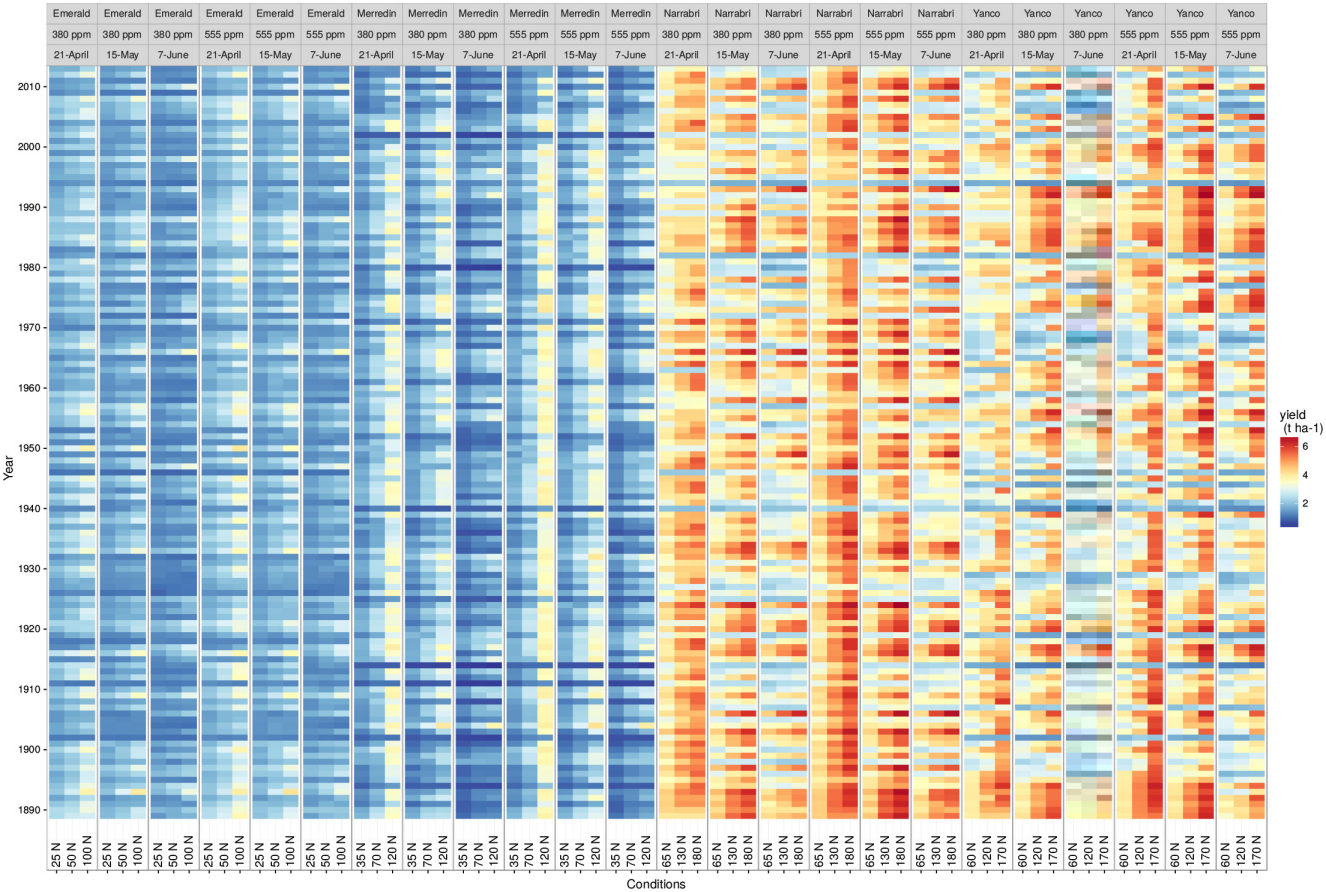
Heatmap of yield response to climate and management practices in all growing environments studied. Simulated yield for cv. *Hartog* is presented for each sites (Emerald, Merredin, Narrabri, Yanco), *CO*_2_ levels (380 and 555 ppm), sowing dates (21 April, 15 May, 7 June), fertilization (x-axis, potential mineral nitrogen applied before decision model, in kg ha^-1^) and climatic years (y-axis) i.e 9000 growing environments in total.

Four sites were chosen to capture part of the variability in soil types and rainfall patterns that are experienced across the dryland wheatbelt (Table 1; Fig 1). Simulated yield for 1889-2013 reflected these differences in environments, with median yield ranging from 1.72 t ha-1 in Emerald to 4.10 t ha-1 at Narrabri (Fig 3). High inter-annual variability was also simulated and reflected the broad range of water deficits and temperature events that Australian wheat experience across seasons [5, 7].

### About a half of the studied traits had little or no impact on yield in the target population of environments (TPE)

A global sensitivity analysis was performed to get a general picture of the effect of APSIM-Wheat parameters on yield response in the TPE. While the results from the sensitivity analysis strongly depend on the ranges of variation for the input traits, such ranges are scarcely available for all the considered traits despite numerous studies and reviews giving informative indications of partial genetic ranges for some traits [47–50]. To perform a broad screen of parameters, the sensitivity analysis was done with variations of ± 20% from the reference value (*Hartog* cultivar) of each parameter (S1 Table), except for some function parameters for which variations were adapted to increase the biological likelihood of the results (see S1 Fig). Another analysis was conducted with variation of ± 50% to test a broader range of variation, but this led to a high proportion of crop failure, due in particular to excessive senescence (data not shown).

**Fig 4:**
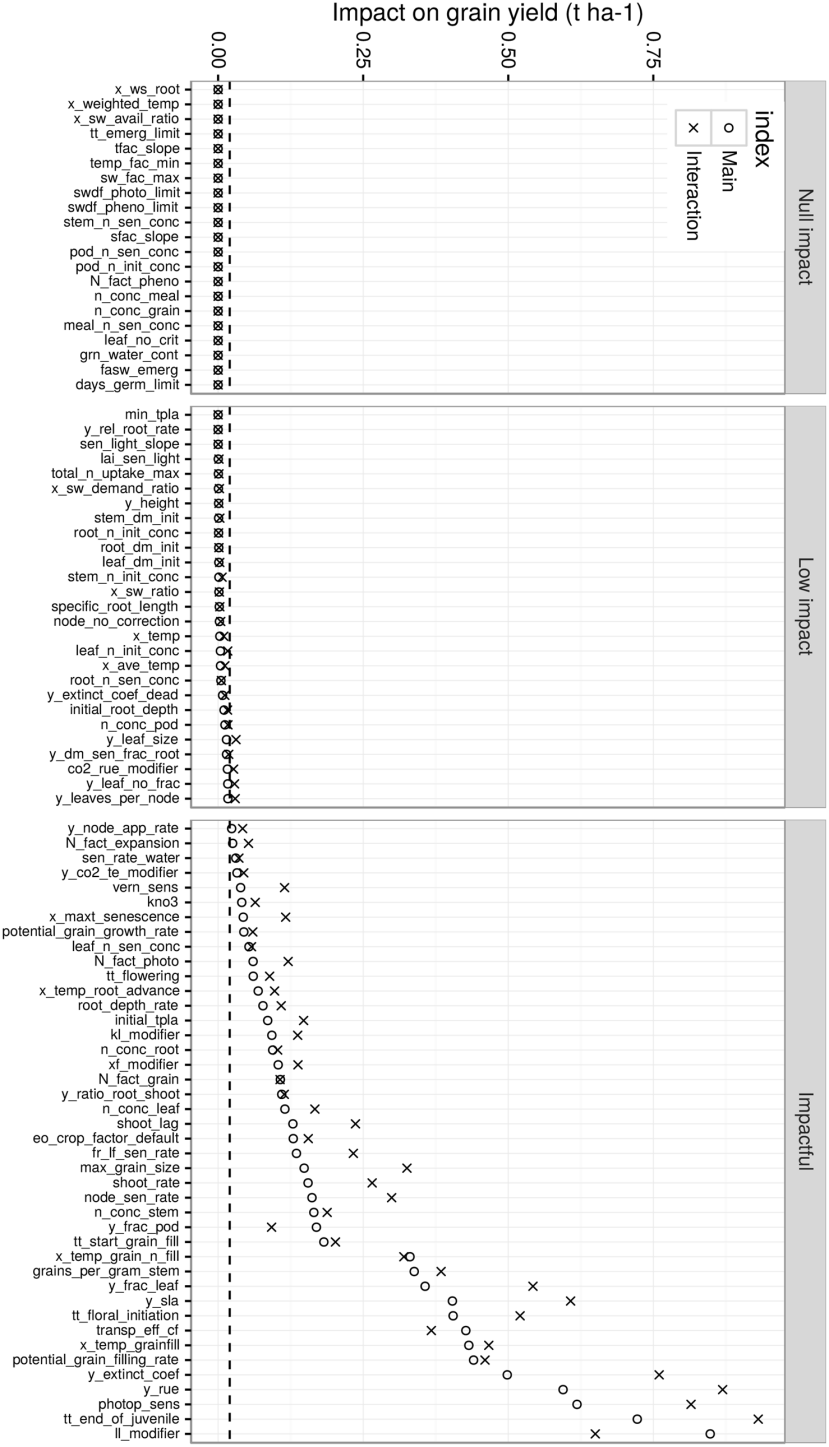
Screening for influential traits in the TPE (control conditions). Traits were ranked by increasing mean main sensitivity index and were grouped into three groups (panels): *null impact*, *low impact* and *impactful* group. Note that all impacts are positive, as given by the sensitivity analysis method. A description of traits is presented in S1 Table. Concerning sensitivity indexes, the main effect (circle) is an estimation of the linear influence of the considered trait on grain yield, while the interaction effect (cross) is an estimation of non-linear and/or interaction effect(s) of the trait. The horizontal dashed line corresponds to the 20 kg ha^-1^ threshold above which traits are considered as impactful.

About half of the studied traits (48/90) were not or only weakly impacting yield (average effect of less than 20 kg ha^-1^) in the TPE (Fig 4). Among those traits, 21 had no impact on yield or any other of the studied output variables (i.e. flowering, maturity, LAI, biomass, grain number, size and protein) in any environments. Two options could explain such null impacts: (1) the parameter corresponding to the trait simply did not have any role in the model algorithm for wheat (some parameters are only used for other crops in the APSIM framework) or (2) the traits were influential only in agricultural conditions other than tested here (e.g the sum of temperature until emergence failure, *tt_emerg_limit*).

The other 27 traits showed a weak mean impact on yield (< = 20 kg ha-1) in the TPE, often because the conditions required to induce a substantial impact are rarely encountered. This group included traits that may have been considered as important a priori, such as potential leaf area (*y_leaf_size*) or maximum temperature for thermal-time accumulation (*x_temp*).

Traits with a mean impact on yield of > 20 kg ha-1 were considered in more detail (42 traits; Fig 4). Overall, 29 traits had a mean impact between 20 and 25 kg ha-1, eight traits had an impact between 25 and 50 kg ha-1, and only five traits had a mean impact greater than 50 kg ha-1. The five most influential traits in terms of both mean and interaction effects ($\mu_{i}^{*}$ and *σ*_*i*_) in the tested conditions were: the water extractability by roots (*ll_modifier*), the thermal time required to reach floral initiation (*tt_end_of_juvenile*), the photoperiod sensitivity (*photop_sens*), the radiation use efficiency (*y_rue*), and the radiation extinction coefficient (*y_extinct_coef*).

Among the 42 influential traits, only a few showed a linear impact on yield, i.e. their main effect was greater than their interaction effect, e.g. the fraction of biomass partitioned to the spike rachis (*y_frac_pod*), the water extractability by roots (*ll_modifier*), the wheat coefficient for transpiration efficiency (*transp_eff_cf*) and the temperature effect on grain demand (*x_temp_grain_fill*). Most of the influential traits had a ratio of interaction:main effect between 1 and 1.8, denoting either a large non-linear effect or an effect largely influenced by other traits. Traits such as senescence-related traits and grain potential biomass (*max_grain_size*) had higher ratio (> 1.8).

### Several traits had a strong impact on physiological processes related to phenology, biomass and grain production

**Fig 5:**
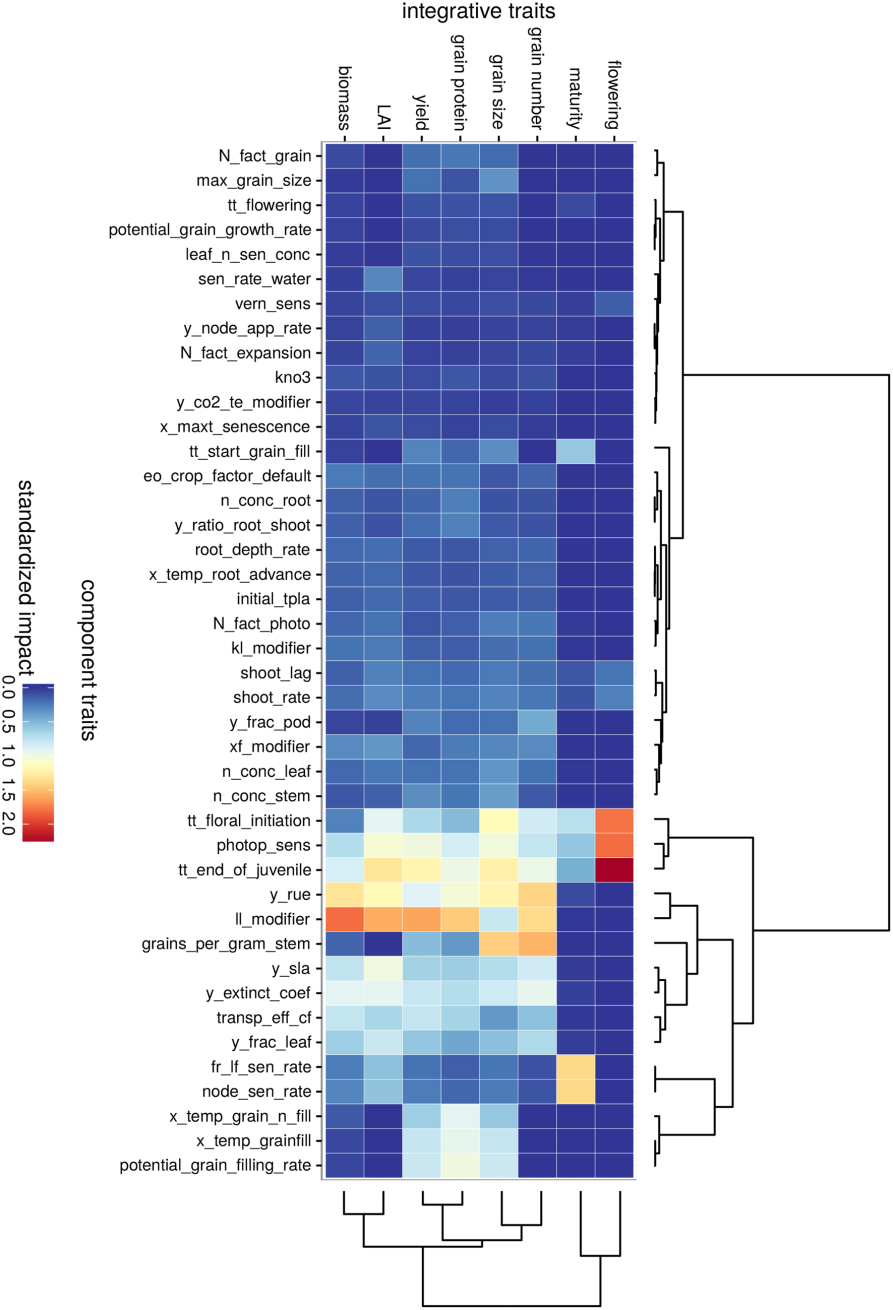
Overview of APSIM-Wheat sensitivity to trait modification. The heatmap shows the impact (positive in the Morris method) of selected component traits (model inputs, x-axis) modification on integrated traits (model outputs, y-axis). Component traits (top dendrogram) and integrated traits (right dendrogram) were ordered with hierarchical clustering based on the similarities among impacts. Trait impact was standardized to be comparable across integrated traits (model output variables).

To better understand the effects of plant traits in the TPE, the 42 influential component traits were clustered based on their main effect on eight integrated traits related to phenology, leaf area, and nitrogen and carbon accumulation and partitioning (Fig 5). Component traits were mainly clustered in three groups (dashed line in Fig 5): lesser influential traits, traits that strongly impacted all outputs, and traits that strongly impacted a subset of integrated traits.

Overall, crop phenology (flowering and maturity time) was mostly affected by six component traits (thermal time from emergence to floral initiation, from floral initiation to flowering and to a lesser extent from flowering to the beginning of grain filling; photoperiod sensitivity and two leaf senescence traits), while the remaining traits had little to no impact. Traits affecting grain-filling (*x_temp_grain_n_filling*, *x_temp_grainfill*, *potential_grain_filling_rate*) were clustered together, and had a high impact on grain size, grain protein and yield. On the other hand, about another 10 traits were found to substantially impact leaf area, biomass and grain production. As may be expected, the water extractability (*ll_modifier*), which affects the maximum amount of soil water that can be extracted, impacted traits such as LAI at flowering, biomass at maturity, grain number and yield. The trait *grains_per_gram_stem* which relates to the potential of the crop to set grains based on its carbon status (proportional to stem weight at flowering), affected grain number but had a relatively little impact on yield given trade-offs on grain size in these largely water-limited environments.

Globally, the impact pathway of traits on physiological processes reflected the sub-component of the crop model where parameters were involved.

### Impacts of influential traits were strongly dependent on environmental and management conditions

**Fig 6:**
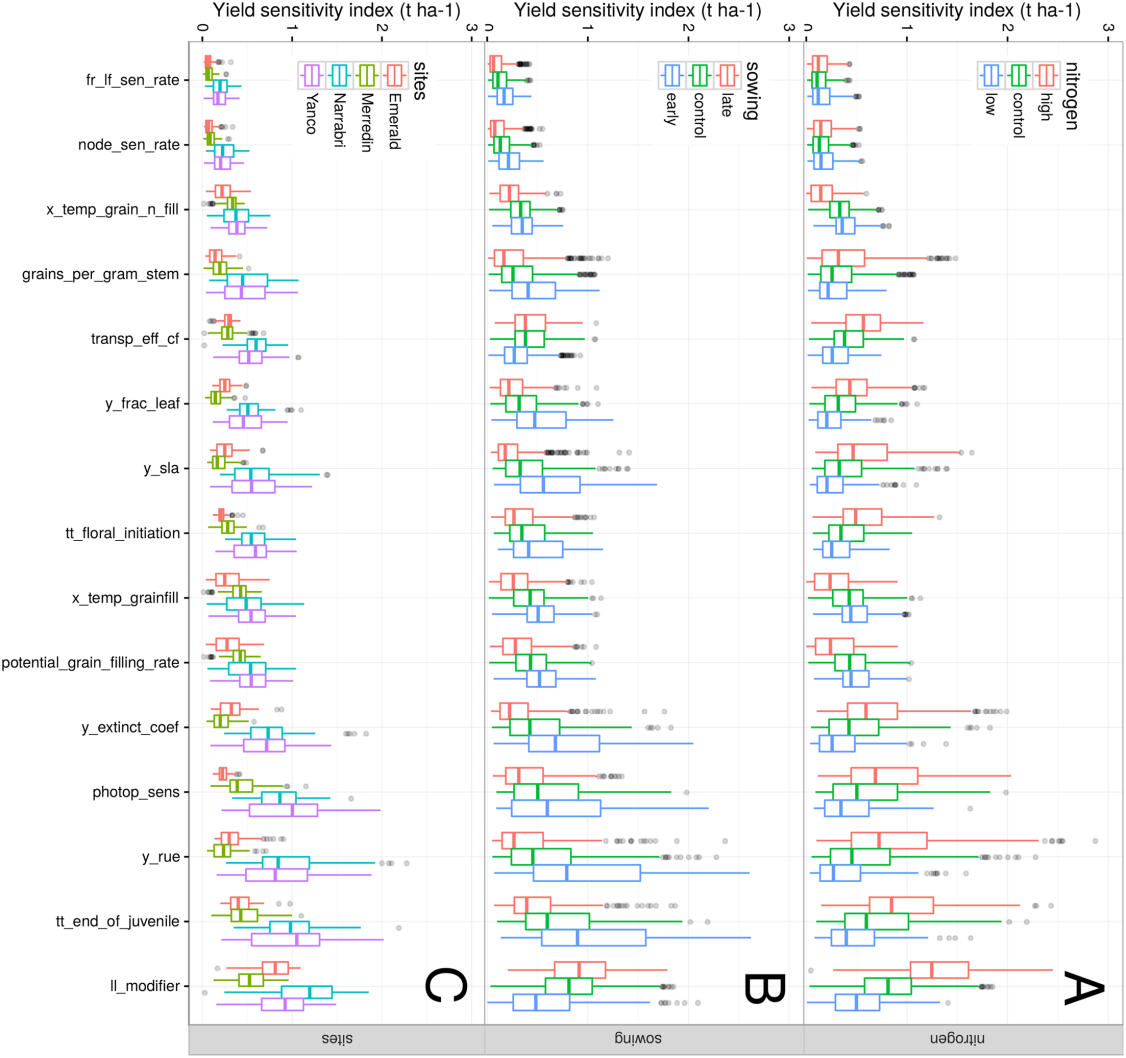
Yield sensitivity to a variation of selected influential trait. Trait main impacts were calculated from a sensitivity analysis and are presented for different nitrogen treatments (A), sowing dates (B) and sites (C) and in the TPE (control conditions) unless mentioned (i.e. high/low nitrogen, early/late sowing).

The variability of trait impacts arose from high trait × environment interactions (Fig 6), i.e. the modification of a trait did not result in the same change in output trait depending on the growing conditions. Main yield impacts of individual component traits ranged from 0.02 t ha-1 (screening threshold) to 2.87 t ha-1 (potential radiation use efficiency, *y_rue*, under high nitrogen conditions).

Most traits had a larger yield impact when management practices and climatic conditions were “non-limiting”, e.g. high fertilization, high soil water holding capacity (Yanco, Narrabri) and early sowing (i.e. long cropping season). By contrast, response traits (e.g. *x_temp_grain_fill*, *transp_eff_cf*) impacted yield in more extensive conditions (e.g. low nitrogen). For instance, water extractability by roots (*ll_modifier*) had more impact for late-sown than for early-sown crops, as such crops are more prone to drought.

### Identification of influential traits with low dependence to climate uncertainty

**Fig 7:**
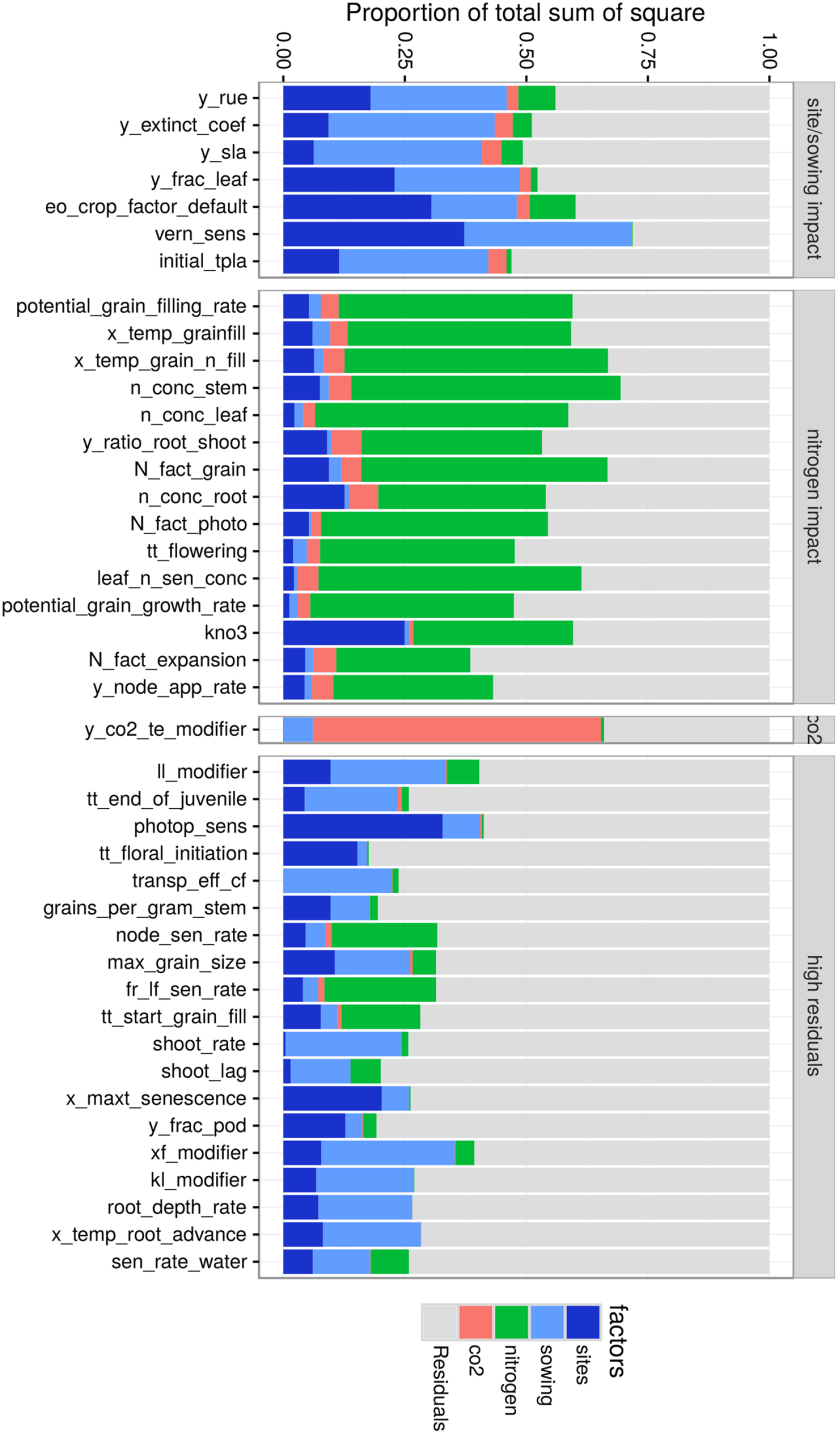
Variance components of trait main impact for major environmental factors. For each influential trait, the proportion of variance explained by environmental factors (site, nitrogen fertilization, sowing date and *CO*_2_ level) was calculated in an ANOVA on simulated yield for crops in the 9000 studied growing conditions. Traits were clustered in groups based on the proportion of explained variance by environmental factors (horizontal panels). Cluster identified corresponded to traits mainly impacted by site and sowing date (first panel), nitrogen fertilization (second panel), CO2 (third panel) and traits having a high residual component (fourth panel).

The variance of trait impacts on yield across the 9000 studied environments was partitioned for each studied traits into four controllable environmental factors (*site*, *sowing date*, *nitrogen fertilization* and *CO*_2_ *level*) and one uncertainty-related factor (*residuals*) that aggregated the factor *year*, the interaction among “controllable” factors and the residuals (Fig 7). Despite the coarseness of the approach and the fact that trait main impacts were only considered as absolute value (no distinction between negative and positive impact on yield), traits with both a strong mean impact and an impact variability that mainly depends on “controllable” factors would potentially be easier for consideration for breeding.

Traits were *a posteriori* clustered in four groups (horizontal panels in Fig 7), which can be described as: (1) site/sowing impact, which may be related to water or temperature driven processes, (2) nitrogen impact, (3) *CO*_2_ impact and (4) high residuals (uncertainty). Traits in the site/sowing, nitrogen and *CO*_2_ groups displayed both high and relatively stable main impact on yield. The nitrogen-impact group included all studied traits related to grain filling, indicating that modifications of such traits could reliably impact yield providing adequate nitrogen fertilization. On the other hand, the site/sowing-driven group included traits such as the potential radiation use efficiency (*y_rue*), the light extinction coefficient (*y_extinct_coef*) and the potential leaf surface area (*y_sla*), which may be linked to the available water resources or thermal regime (e.g. short/long crop cycle). Traits in the *high residuals* group were influential but not stable, meaning that a modification of such traits did not yield the same return depending on years and/or due to interaction with other traits. Phenology-related traits (*tt_end_of_juvenile*, *tt_floral_initiation*) and water extractability by roots (*ll_modifier*) displayed such behavior, indicating that impact was likely linked to the level of environmental resources available (water or temperature, in this case), which is expected in these types of environments.

This variance analysis also highlighted expected trait × environment interactions. For instance, a high *CO*_2_ concentration triggered the impact of the *CO*_2_ response on transpiration efficiency (*y_co2_te_modifier*). Note that the effect on radiation use efficiency (*co2_rue_modifier*) was not identified as influential in the TPE (i.e. when no change in *CO*_2_; Fig 4) and was thus not included in the further analysis. Also, photoperiodic and vernalization sensitivities (*photop_sens*, *vern_sens*) had contrasting effect across sites and sowing dates. These results are consistent with field observations [21].

### Trait impacts were related to the availability of environmental resources

**Fig 8:**
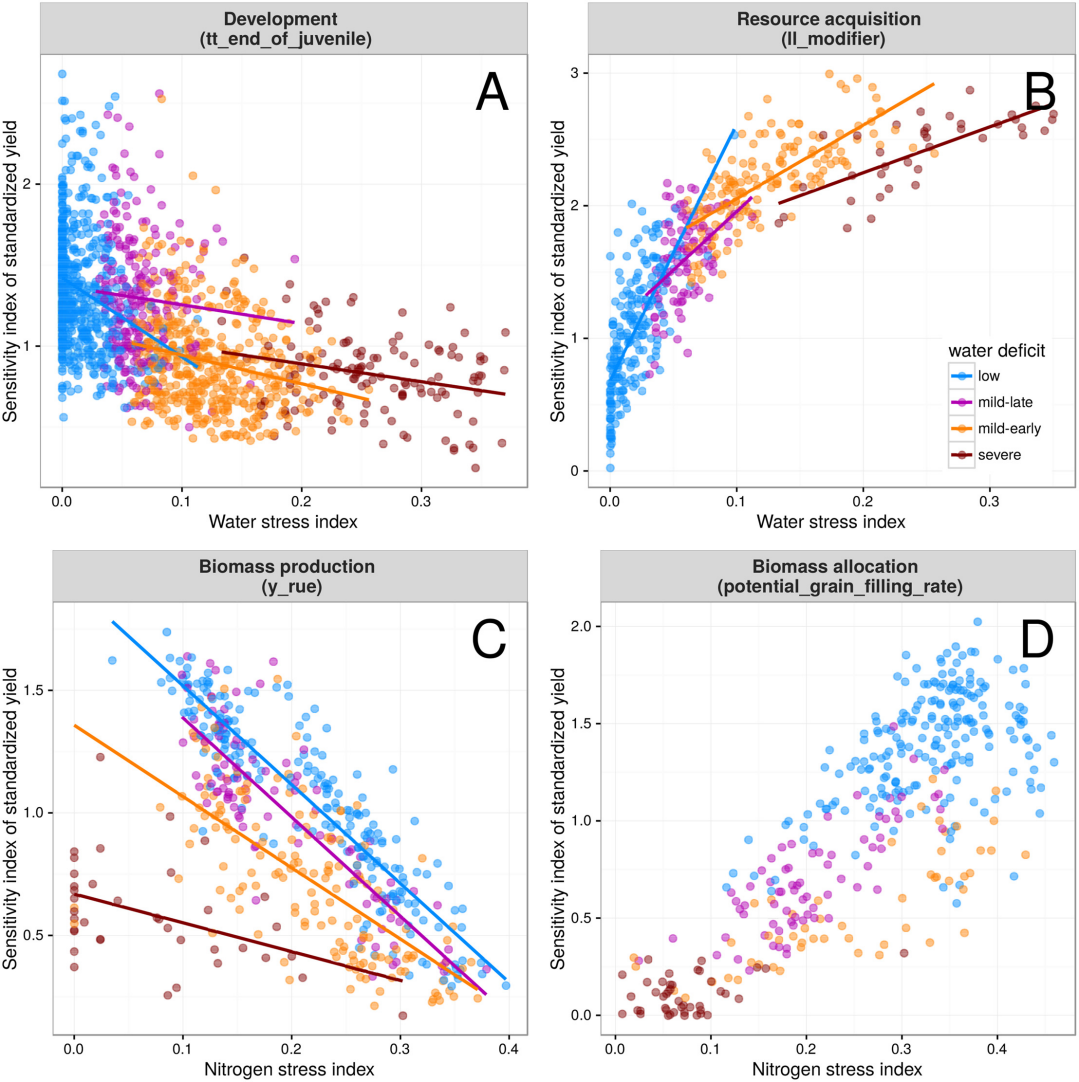
Sensitivity index of standardized yield for selected component traits involved in crop development (A), resource acquisition (B), biomass production (C) and biomass allocation (D) relative to seasonal water- or nitrogen-stress indices. Yield impact was assessed for the thermal time required to reach floral initiation (*tt_end_of_juvenile*), the water extractability by roots (*ll_modifier*), the radiation use efficiency (*y_rue*), and biomass allocation to grains (*potential_grain_filling_rate*). As sensitivity indices are computed independently for each condition (combinations of sites × year × management), a standardized sensitivity index was used to allow comparison of indices across environments. In this case, simulated yield was standardized ($x^{\prime }=\frac{x-mean(x)}{sd(x)}$) within each of the 9000 environment conditions before computing elementary effects and sensitivity indices (which are always positive in Morris method). The water-stress index [7] indicates the degree to which the soil water extractable by roots (water supply) is able to match the potential crop transpiration (water demand). The nitrogen-stress index is a factor computed by APSIM that determines limiting N level affecting leaf photosynthesis [29]. Both indexes ranged from 0 (no-stress) to 1 (extreme stress). Data are presented for representative drought-pattern environment types (colors), namely “low” (ET1) with stress-free or short-term water-deficits; “mild-late” (ET2) with mild water shortage mainly occurring during grain filling; “mild-early” (ET3) with severe water stress starting during the vegetative stage and relieved during mid-grain filling; and “severe” (ET4) with water deficit from early stages throughout the grain-filling periods [7]. Lines represent linear regressions fitted by environment types.

Strong interactions were identified between environmental factors and trait impact on yield (Fig 8) for several traits involved in plant development (*tt_end_of_juvenile*), resource acquisition (*ll_modifier*), biomass production (*y_rue*) and biomass allocation (*potential_grain_filling_rate*) processes. Computed seasonal stress indices for water and nitrogen (see caption of Fig 8) were used to highlight these dependencies between environmental stress and the impact resulting from a trait modification. Modifications in phenology (*tt_end_of_juvenile*) impacted yield the most in wet environments (stress index near zero), when yield potentials were the greatest (Fig 8A). Nevertheless, this trait had substantial impacts in all environments, including the most severely water limited. Change in water extractability by roots (*ll_modifier*) also responded to water deficit (Fig 8B) with maximum impacts in severe water deficits. Impacts were slightly less important in mid-early water deficits. They rapidly decreased in less stressed conditions, but remained substantial. Modifications in potential photosynthesis (*y_rue*) had impacts related to both water and nitrogen availability (Fig 8C). The relation between impact and nitrogen availability was linear within each drought environment type, and the slope of the relation decreased with the severity of the water deficit (i.e. the impact response to N was greater in non-limiting water conditions). Modifications in biomass allocation to grains (*potential_grain_filling_rate*) led to maximum yield impact in low water deficit (Fig 8D) and in severe nitrogen deficits. Yield impact was increasing with nitrogen deficit but showed a weaker linear correlation in conditions with severe nitrogen stress (r = 0.47).

## Discussion

### An *in silico* method to search for potential candidate traits for breeding

Environmentally-adaptive traits do not scale well from molecular-, organ- or plant-level to the crop level, particularly when targeting yield under stressful conditions [2, 51–53]. This difficulty in demonstrating and estimating the impacts of traits across scales potentially limits inference of trait value, and is partly responsible for the non-integration of physiological progress in breeding programs.

Here, the problem was approached in the opposite direction (top-down), to unravel the phenotypic plasticity observed in complex traits into individual trait contribution at the crop level. Process-based crop models are designed to integrate physiological processes and their impact on the local environment (e.g. soil water uptake) based on parameters reflecting plant traits (parameterization), environmental factors and management inputs. As a result, such models simulate genotype × environment interactions and estimate integrated traits (e.g. yield) as emergent properties [1, 2, 22, 54, 55]. Here, the APSIM-Wheat model, which has been widely tested for Australian conditions was used to weigh the impact of numerous plant traits across the Australian wheatbelt, taking into account climatic variability, trait × trait interactions and trait × environment interactions.

While APSIM-Wheat has over 500 parameters with 103 identified as potentially varying with genotype, the approach proposed in this paper allowed the identification of 42 influential traits in the target population of environments (TPE; Fig 4). Of these 42 traits, 23 had an impact that was relatively stable, meaning that the variance of their impact was explained by “controllable” factors (i.e. site, sowing date, nitrogen fertilization and CO2 level) more than those dependent on climate uncertainty.

Overall, the screening phase (sensitivity analysis) allowed the identification of the most influential traits for yield (Figs 4–6); and the searching phase (variance analysis, relation with specific environmental factors) gave indications as to which traits to target when considering different types of environments within this sample of Australian environments, e.g. high vs low N conditions; Figs 7 and 8. Such an approach could thus help in estimating trait scalability, and give a form of return on investment with an estimation of expected gains from trait modifications. However, additional knowledge is required when considering the potential value for crop breeding (e.g. degree of genotypic variability that may exist for these traits, trait heritability).

### Potential candidate traits for improving yield in the Australian wheatbelt

Based on the APSIM-Wheat simulations and a global sensitivity analysis, traits relative to phenology (*tt_end_of_juvenile*, *photop_sens*, *tt_floral_initiation*), resource acquisition (water extraction, *ll_modifier* and light interception, *y_extinct_coef*), resource use efficiency (*y_rue*, *transp_efficiency_coef*) and biomass allocation to the grain (*potential_grain_filling_rate*, *grains_per_gram_stem*) were among the most important traits in the TPE, assuming a “genetic” variation of ± 20% around trait value of the reference cultivar *Hartog* (Fig 4). It is important to keep in mind that the results of a sensitivity analysis strongly depend on the chosen range of trait variation, and that the ± 20% trait variation used in this study under-estimated existing variations for some traits (e.g. *vern_sens*) while it may have over-estimated unknown variations in others. However, assuming that (1) the APSIM model behaves relatively linearly (interaction:main effect ratio of 1-1.5 for most parameters, Fig 4) and (2) new interactions do not arise from this extension in the parameter ranges, a moderate change of this range would not have strongly affected the estimated impacts (main effect), i.e. the most influent traits have correctly been identified in this study. Overall, the approach allowed a first screening of a wide range of traits for which the range of genetic variability is unknown. This work could be improved in the future through the incorporation of knowledge on genetic variability of selected traits. Adjustments on the TPE could also be investigated, for instance, TPE for future climate scenarios could be explored to identify potential traits of future importance, providing crop models can properly deal with these future conditions.

The most important trait in terms of impact on yield was the water extractability by roots (*ll_modifier*; Figs 4 and 5), especially in Narrabri and Yanco, which had heavy deep soils and thus a high water-holding capacity (Fig 6). Genotypic variation in water extractability at depth was observed in root chambers by Manschadi et al. [50], who assessed that this trait could bring about an extra 50 kg ha-1 for every mm of water extracted during the grain filling period, for crops grown in the north-eastern part of the wheatbelt (i.e. ability to extract more water late in the season has a high marginal value in terminal stress environments). Compared to other root-related traits, Veyradier et al. [56] found that this trait was a strong candidate for breeding purpose in terms of potential impact. Field experiments for two cultivars with contrasting water extractability at depth also highlighted the potential of this trait to improve yield in drought-prone conditions [50, 57], which agrees with the increasing yield impact simulated for increasing drought severity (Fig 8B).

Several traits involved in wheat development were identified as playing a major role in crop performance in the TPE (Figs 4–6). Traits related to phenology are usually considered as the primary means to adapt crops to their growing environments [58, 59]. Recently, an association mapping study [60] focused on three traits (earliness *per se*, photoperiod sensitivity and vernalization requirement), whose corresponding parameters in APSIM-Wheat model (*tt_end_of_juvenile*, *photop_sens* and *vern_sens*, respectively) were ranked among the most influential ones in this study (i.e. average main impacts on yield respectively of 0.72, 0.62 and 0.04 t ha-1), despite the fact that our reference cultivar (Hartog) has a low vernalization requirement (*vern_sens* of 1.5). These three traits were found to vary in the ranges of 515-980°Cd, 0-4.1 and 0-2.9 respectively for a broad range of Australian cultivars [21], which is substantially greater than the range tested here (444-666°Cd, 2.4-3.6 and 1.2-1.8), especially for the vernalization requirement. The relative importance of those traits on yield is expected to change when changing their range of variation. In particular, *vern_sens* is expected to have a greater impact in the TPE, as found by Zhao et al. [36] who tested a range of 0-5 for this trait in a similar analysis. Also, unsurprisingly, these three traits were found to be strongly dependent on the site and sowing date (Fig 7) but had a high level of variations (high residuals in Fig 7), which is likely related to interactions with stresses.

Other traits had a strong impact on yield. The most important of these include: (1) the potential RUE (*y_rue*) which is a major target for current research projects aiming to improve photosynthesis efficiency [61–64], (2) plant architecture (*y_extinct_coef*) which has been of interest to some breeders (e.g. durum-wheat CIMMYT) who have selected for erect wheat genotypes [65], and (3) the potential grain filling rate (*potential_grain_filling_rate*), which may be improved by the current efforts of breeders and pre-breeders selecting for stay-green phenotype [66–68], cooler canopy temperature [59, 69, 70], greater reserve remobilisation [71, 72] and/or greater spike photosynthesis [73, 74].

### The importance of properly considering the target population of environments

Depending on the environment/management conditions considered, the ranking of trait main impacts varied across traits (Fig 6), thus highlighting the need to appropriately consider trait effects across the target populations of environments [75]. For instance, the sensitivity to photoperiod (*photop_sens*) had a small impact in Emerald but an important impact in Narrabri and Yanco (Fig 6C). Hence, while most influential parameters by Zhao et al. [36] were also identified as the most influential subset in our study, the discrepancies in trait impact between these two studies partly rose from differences in conditions considered (e.g. sowing dates, fertilization, plant density). Our study also explored climate change impacts on the 42 influential traits and indicated that traits of most value may change in the future, as illustrated for the impact of transpiration-efficiency response to *CO*_2_ (*y_co2_te_modifier*) under different levels of *CO*_2_ (Fig 7). Note that other traits such as radiation-use-efficiency response to *CO*_2_ (*co2_rue_modifier*), which had only minor impact in current climates (Fig 4) and were thus not studied in detail, are likely to have a substantial impact in the future.

### The importance of considering trait combinations rather than single traits

Sadras and Richards [52] argued and illustrated how indirect breeding methods often fail to improve yield not because yield is complex, but rather because those methods do not account for the proper levels of organization, time scales and interactions among traits and with the environment. Similarly, trait impacts in crops subjected to multiple stresses (e.g. nitrogen and water limitation) are rarely considered in traditional physiological approaches [52]. Working with an integrative crop model, we illustrated in this paper how the potential value of traits, in combination with others and for a specific TPE, can be assessed *in silico* by testing (1) whether the trait is likely to impact crop performance (e.g. estimation of main sensitivity index), (2) if this impact is modified by controllable (management) or uncontrollable (climate, genotype × environment interactions) factors, and (3) how the trait impact is distributed among environment-type of importance for the TPE [7].

The systematic presence of interaction effects found with the sensitivity analysis (Fig 4) illustrated that trait interactions are common. Such results highlight the importance of focusing on collections of traits rather on individual traits [76].

Furthermore, although of high importance, links among carbon, water, and nitrogen transfers within crops are experimentally difficult to assess due to their genetic, physiologic, and agronomic complexities. For instance, efficiencies in water- and nitrogen-use can be either unrelated, positively (synergy) or negatively (trade-off) related depending on the environment, the genotype, the level of organization, and the time scale at which such efficiencies are defined [52, 77, 78].

Overall, the complexity of crop systems highlights the potential benefit of using modeling approaches. Together with genetic criteria (e.g. availability of genetic variability, pleiotropy and heritability) and technical criteria (rapid, cost-effective, and reliable phenotyping), model-based approaches (assuming the relevance of the process-based model, of the genetic range tested and of the TPE) could help breeding to improve crop performance under changing environments [2, 52].

### A tool to overview and improve crop models

From a modeling point of view, crop models are evolving over time, while physiological knowledge underlying crop functioning gradually improves. Model improvements are thus regularly performed with algorithm modifications being tracked over time. However, the effects of such modifications on the model-prediction capacity are usually not clearly documented nor shared among all model users and developers. Hence, with different developers focusing simultaneously or successively on a model, there is a high risk of developing increasingly complex and harder to understand algorithms. Problems caused by this increased complexity may affect the quality of the model, but may be revealed and addressed by using exploration methods throughout model-development phases to visualize the in-progress modeling state. Systems analyses, as done in this paper can for instance enable developers to quickly assess changes in model response due to variation in specific processes, and notice potential problems.

In this study, we attempted to consider the maximum proportion of traits utilized in APSIM-Wheat. The use of function-table parameters in APSIM complicated the estimation of the total number of values used as parameters and the assessment of individual parameter impact on output variables. Overall, about half of the plant-related parameters of APSIM-Wheat had no impact, keeping in mind that those parameters may be useful for other crops, or other processes (e.g. responses to high-temperature or soil minerals). While using a global sensitivity analysis to identify such parameters may appear as an excessive method, the computational cost to include all parameters (with null, low or high impact) was lower than the time and expertise needed to analyze the source code and manually identify subsets of parameters, in the case of this complicated crop model.

In total, 42 parameters were identified as influential, as they had an average main impact greater than 20 kg ha-1 in the TPE. However, only 5 parameters had a mean impact greater than 50 kg ha-1. Martre et al. [76] proposed physiological reasons to explain such a surprisingly low number of influential parameters in crop models: (1) number of trade-offs occur with traits often having compensating effects when scaling up from plant to crop level (e.g. once canopies are well established, increasing the leaf surface area may not improve light interception and thus photosynthesis) and (2) the fact that complex characters such as grain yield and protein concentration are inherently determined at the population level rather than at the organ or plant level [79]. While model over-parameterization can result from model development as well as model design, indicators can help to track the model complexity and performance. In this context, the use of the exploration methods described here provides an overview of the model global response to perturbation (e.g. Figs 3–5).

Finally, such sensitivity analysis can help to identify traits most important for parameter calibration for cultivars [36]. Such targeted calibration can later be implemented with either frequentist [80] or Bayesian parameter estimation algorithms [81].

## Conclusion

Phenotyping and breeding strategies can be improved by better understanding the yield-trait performance landscapes [82]. We performed a Morris global sensitivity analysis on the APSIM-Wheat model to assess the impact of 90 physiological traits on yield for Australian rain-fed wheat crops. The genotype × environment × management (G×E×M) landscape was explored using 82 million individual simulations for the target population of environments (TPE), combining a factorial design for the environment × management effects and a Morris sampling design for APSIM trait parameters. Our analysis highlights 42 parameters substantially impacting yield in most of the TPE. Among those, a few parameters related to phenology, resource acquisition, resource use efficiency and biomass allocation were identified as potential candidates for crop improvement.

As a final conclusion, the integration of G×E×M interactions through modeling approaches is an increasingly topical consideration to help prioritizing investments of research efforts for the benefit of breeding [17]. However, newly-gained computational knowledge has to be constantly confronted to physiological reality in order to determine the complexity of G×E×M interactions that impede progress in crop productivity.

## Supporting Information

S1 Fig Range of variation used for function parameters. Each graph represents one function parameter (*x* and *y* vectors), except for grouped parameters (i.e. leaf, stem and pod nitrogen demand). The graph titles match the *Process* column in S1 Table. Nominal values are indicted in green, while minimum and maximum values are displayed blue and red, respectively. As some parameters were grouped to be modified together, different symbols are used for related processes (maximum, critical and minimum nitrogen content) as defined in APSIM-wheat [29].
(PDF)

S1 Table Description of the APSIM-wheat parameters included in the sensitivity analysis. *Module* refers to the sub-model where the parameter is used in APSIM-wheat, *Process* refers to the physiological process targeted by the considered parameter and *Factor* is the parameter name used in the present study and in the APSIM documentation [29], where a complete description of the parameters is given. The *Default Value* field lists the nominal value of the parameter for cultivar Hartog in APSIM-wheat 7.5 (only first three values were presented when the parameter is defined as a vector). In the *Process* field, influential parameters in indicated in bold and parameters that were grouped together for physiologic reasons are identified by (*).
(PDF)
