# Supplementary figures and images for: Assessment of the Potential Impacts of Wheat Plant Traits across Environments by Combining Crop Modeling and Global Sensitivity Analysis
Source: PLoS One. 2016 Jan 22;11(1):e0146385. doi: 10.1371/journal.pone.0146385 (PMC4723307; doi:10.1371/journal.pone.0146385)

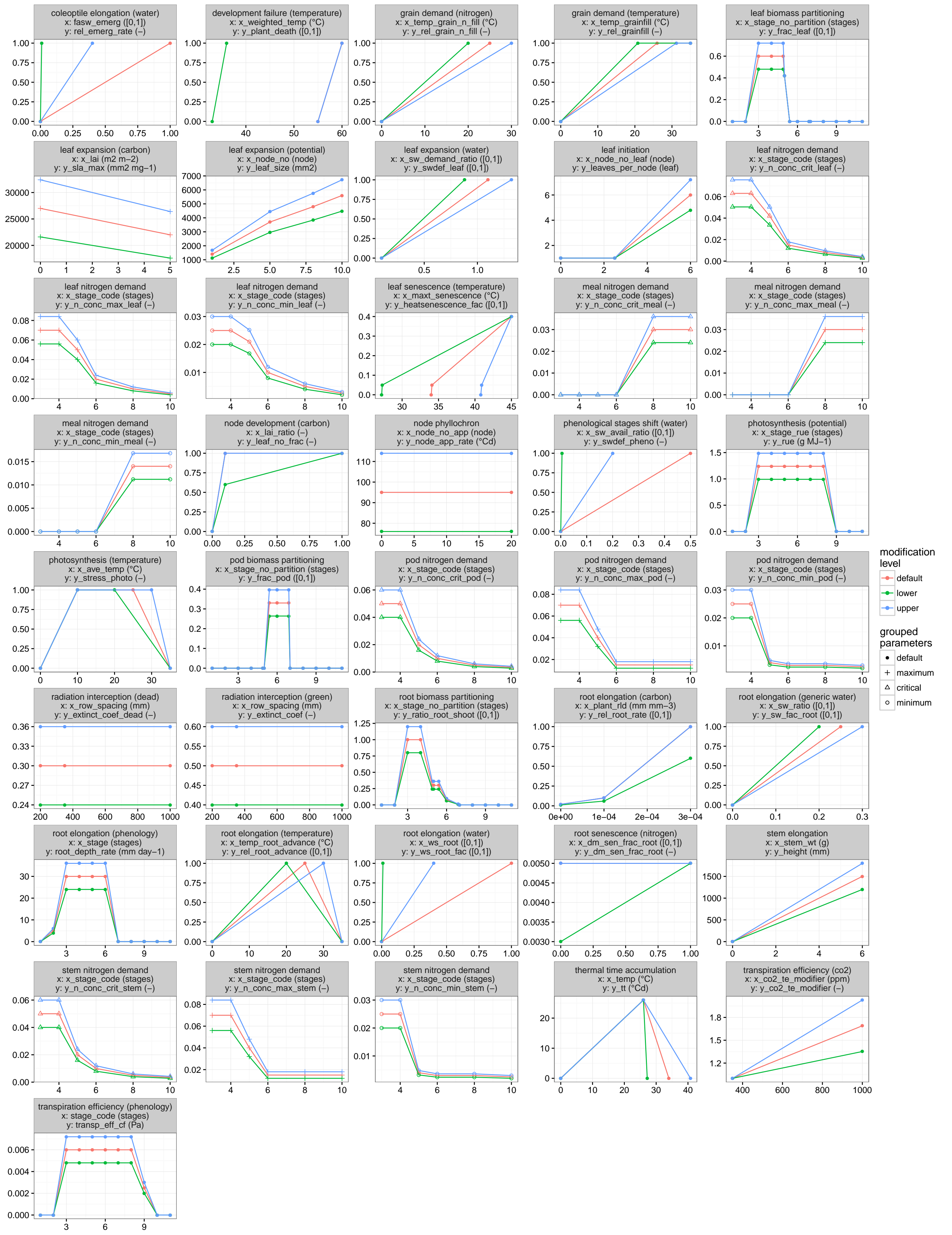

Supplement: S1 Fig — Each graph represents one function parameter (x and y vectors), except for grouped parameters (i.e. leaf, stem and pod nitrogen demand). The graph titles match the Process column in S1 Table. Nominal values are indicted in green, while minimum and maximum values are displayed blue and red, respectively. As some parameters were grouped to be modified together, different symbols are used for related processes (maximum, critical and minimum nitrogen content) as defined in APSIM-wheat [29]. (PDF) [file pone.0146385.s001.pdf]
