# Supplementary material for: Assessment of the Potential Impacts of Wheat Plant Traits across Environments by Combining Crop Modeling and Global Sensitivity Analysis
Source: PLoS One. 2016 Jan 22;11(1):e0146385. doi: 10.1371/journal.pone.0146385 (PMC4723307; doi:10.1371/journal.pone.0146385)
Supplement: S1 Table — Module refers to the sub-model where the parameter is used in APSIM-wheat, Process refers to the physiological process targeted by the considered parameter and Factor is the parameter name used in the present study and in the APSIM documentation [29], where a complete description of the parameters is given. The Default Value field lists the nominal value of the parameter for cultivar Hartog in APSIM-wheat 7.5 (only first three values were presented when the parameter is defined as a vector). In the Process field, influential parameters in indicated in bold and parameters that were grouped together for physiologic reasons are identified by (*). (PDF) [file pone.0146385.s002.pdf]

Module	Process	Factor	Description	Default Value	Unit
phenology	thermal time accumulation	x_temp	Cardinal temperatures affecting the effective thermal time (y_tt = 0 26 0 oCd)	0 26 34	°C
phenology	germination failure (time)	days_germ_limit	Maximum days allowed after sowing for germination to take place	40	days
phenology	coleoptile elongation (water)	fasw_emerg	Fraction of available soil water affecting the stress factor [0-1] for thermal time calculation between germination and emergence (rel_emerg_rate = 1 1)	0 1	[0,1]
phenology	**emergence (temperature)**	shoot_lag	Time lag before linear coleoptile growth starts	40	°Cd
phenology	**coleoptile elongation (temperature)**	shoot_rate	Degree days required to increase coleoptile by 1 mm	1.5	°Cd mm-1
phenology	emergence failure (temperature)	tt_emerg_limit	Maximum degree days allowed for emergence to take place	300	°Cd
phenology	**node phyllochron**	y_node_app_rate	Thermal time between the appearance of successive nodes as a function of the node number of the main stem (x_node_no_app = 0 20)	95 95	°Cd
phenology	leaf initiation	y_leaves_per_node	Leaf per node defined as a function of x_node_no_leaf=0 2.5 6	1 1 6	leaf node-1
phenology	node development (carbon)	y_leaf_no_frac	Leaf appearance depending on LAI (x_lai_ratio = 0 0.1 0.8 1)	0 1 1	[0-1]
phenology	phenology failure	swdf_pheno_limit	Critical cumulative phenology water stress, above which the crop fails	25	-
phenology	development failure (temperature)	x_weighted_temp	Temperature affecting plant death (y_plant_death = 0 1)	55 60	°C
cultivar	**phenological stages shift (photoperiod)**	photop_sens	Sensitivity to photoperiod	3	-
cultivar	**phenological stages shift (vernalisation)**	vern_sens	Sensitivity to vernalisation	1.5	-
cultivar	**thermal time accumulation (juvenile)**	tt_end_of_juvenile	Thermal time between plant emergence and end of juvenile stage	400	°Cd
cultivar	**thermal time accumulation (floral initiation)**	tt_floral_initiation	Thermal time between end of juvenile and floral initiation	555	°Cd
cultivar	**thermal time accumulation (flowering)**	tt_flowering	Thermal time between floral initiation and flowering	120	°Cd
cultivar	**thermal time accumulation (grain filling)**	tt_start_grain_fill	Thermal time between flowering and start of grain filling	545	°Cd
cultivar	stem elongation	y_height	plant canopy height for stem weight per plant (x_stem_wt = 0 6 g plant-1)	0 1500	mm
cultivar	**grain development**	grains_per_gram_stem	Number of grains that are set depending on the stem dry weight	25	grain g-1
cultivar	**grain demand (flowering)**	potential_grain_growth_rate	Potential rate of grain growth from flowering to grain fill	0.001	g d-1
cultivar	**grain demand (maturity)**	potential_grain_filling_rate	Potential rate of grain growth during grain filling	0.002	g d-1
cultivar	**grain demand (maximum)**	max_grain_size	Potential grain size	0.041	g
biomass	initial leaf biomass	leaf_dm_init	Initial leaf dry matter	0.003	g
biomass	initial root biomass	root_dm_init	Initial root dry matter	0.01	g
biomass	initial stem biomass	stem_dm_init	Initial stem dry matter	0.0016	g
biomass	photosynthesis (co2)	co2_rue_modifier	Factor affecting the CO2 compensation point	163	-
biomass	biomass growth (generic temperature)	tfac_slope	Temperature stress factor slope	0.0125	-
biomass	biomass growth (generic temperature, min)	temp_fac_min	Temperature stress factor minimum	0.69	-
biomass	biomass growth (generic water)	sfac_slope	Soil water stress factor slope	-0.125	-
biomass	biomass growth (generic water, max)	sw_fac_max	Soil water stress factor maximum	1.125	-
biomass	biomass growth (temperature)	x_ave_temp	Daily mean temperatures affecting the RUE multiplier [0-1] (y_stress_photo = 0 1 1 0)	0 10 25 35	°C
biomass	**radiation interception (green)**	y_extinct_coef	Extinction coefficient of green leaves as a response to row spacing (x_row_spacing = 200 350 1000 mm)	0.5 0.5 0.5	-
biomass	radiation interception (dead)	y_extinct_coef_dead	Extinction coefficient for dead leaves as a response to row width (x_row_spacing = 200 350 1000 mm)	0.3 0.3 0.3	-
biomass	**grain demand (temperature)**	x_temp_grainfill	Temperatures affecting stress index [0-1] for the potential grain filling rate (y_rel_grainfill = 0 1 1)	0 26 35	°C
biomass	**grain demand (nitrogen)**	x_temp_grain_n_fill	Temperatures affecting stress index [0-1] for potential N filling rate (y_rel_grain_n_fill = 0 1 1)	0 25	°C
biomass	**photosynthesis (potential)**	y_rue	Radiation Use Efficiency depending on stages	0 0 1.24 1.24 1.24 1.24 1.24 1.24 0 0 0	g MJ-1
biomass	**root biomass partitioning**	y_ratio_root_shoot	Root/shoot ratio as a function of specific stages (x_stage_no_partition = 1 2 3 4 4.9 5 5.4 6 6.9 7 8 9 10 11)	0 0 1 1 0.3 0.3 0.3 0.08 0.01 0 0 0 0 0	[0,1]
biomass	**leaf biomass partitioning**	y_frac_leaf	Fraction of remaining dry matter allocated to leaves for specifc stages (specific stages (x_stage_no_partition = 1 2 3 4 4.9 5 5.4 6 6.9 7 8 9 10 11)	0 0 0.6 0.6 0.6 0.42 0 0 0 0 0 0 0 0	[0,1]
biomass	**pod biomass partitioning**	y_frac_pod	Fraction of dry matter allocated to rachis for specific stages (x_stage_no_partition = 1 2 3 4 4.9 5 5.4 6 6.9 7 8 9 10 11)	0 0 0 0 0 0 0.33 0.33 0.33 0 0 0 0 0	[0,1]
biomass	grain water content	grn_water_cont	Water content of grain	0.125	g g-1

Module	Process	Factor	Description	Default Value	Unit
leaf	**leaf expansion (init)**	initial__tpla	Initial total plant leaf area	200	mm2
leaf	**leaf expansion (carbon)**	y__sla	Parameter added to change minimum and maximum values all at once	1	-
leaf	leaf expansion	node__no__correction	Number of growing leaves in the sheath	2	leaf
leaf	leaf expansion (carbon)*	sla__min	Minimum specific leaf area	18000	mm2 mg-1
leaf	leaf expansion (carbon)*	y__sla__max	Maximum specific leaf area as a function of daily change in LAI (x__lai = __sla__max)	27000 22000	mm2 g-1
leaf	leaf expansion (failure)	leaf__no__crit	Critical number of leaves, below which portion of the crop may die due to water stress	10	leaf
leaf	leaf expansion (potential)	y__leaf__size	Leaf potential size depending on the number of nodes appeared (x__node__no = 1 5 8 10) and the growing leaf number in the sheath (node__no__correction = 2)	1400 3700 4800 5600	mm2
root	**root absorption (capacity)**	ll__modifier	Parameter added to change crop lower limit values for all depths at once	1	-
root	**root absorption (potential)**	kl__modifier	Parameter added to change crop kl values for all depths at once	1	-
root	**root elongation (phenology)**	root__depth__rate	Potential rate of root front increase for the different stages	0 5 30 30 30 30 0 0 0 0 0	mm day-1
root	**root elongation (soil structure)**	xf__modifier	Parameter added to change crop xf values for all depths at once	1	-
root	**root elongation (temperature)**	x__temp__root__advance	Temperatures affecting root front by the [0-1] stress index: y__rel__root__advance = 0 1 0	0 25 35	°C
root	root elongation (carbon)	specific__root__length	Specific root length	105000	mm g-1
root	root elongation (carbon)	y__rel__root__rate	Factor affecting root length density in a layer, which depends on root branching (x__plant__rld = 0 0.0001 0.0003 mm/mm3/plant)	0.02 0.1 1	[0,1]
root	root elongation (generic water)	x__sw__ratio	Water availability affecting the stress factor for root depth growth (y__sw__fac__root = 0 1)	0 0.25	[0,1]
root	root elongation (init)	initial__root__depth	Initial depth of roots	100	mm
root	root elongation (water)	x__ws__root	Water supply demand ratio affecting the increase in root depth (y__ws__root__fac = 1 1)	0 1	[0,1]
water	phenological stages shift (water)	x__sw__avail__ratio	Water availability that affects stress indices for phenology: y__swdef__pheno = 1.0 1.0	0 0.5	[0,1]
water	leaf expansion (water)	x__sw__demand__ratio	Water availability that affects stress indices for leaf expansion: y__swdef__leaf = 0 1	0.1 1.1	[0,1]
water	photosynthesis failure (water)	swdf__photo__limit	Critical cumulative photosynthesis water stress, above which the crop partly fails	99	-
water	**transpiration efficiency (phenology)**	transp__eff__cf	Transpiration efficiency coefficient for aboveground biomass for the different stage codes	0 0 0.006 0.006 0.006 0.006 0.006 0.006 0.0025 0 0	g m-2 mm-1 kPa
water	**water demand (VPD)**	eo__crop__factor__default	Default crop factor for limiting soil	1.5	-
water	**transpiration efficiency (co2)**	y__co2__te__modifier	CO2 factor for transpiration effciency in response to x__co2__te__modifier = 350 700 1000 ppm	1 1.69	-
nitrogen	initial leaf nitrogen	leaf__n__init__conc	(small hypothesis)	0.063	g g-1
nitrogen	initial root nitrogen	root__n__init__conc	Root initial N concentration	0.02	g g-1
nitrogen	initial stem nitrogen	stem__n__init__conc	Stem initial N concentration	0.05	g g-1
nitrogen	initial pod nitrogen	pod__n__init__conc	Rachis initial N concentration	0.063	g g-1
nitrogen	**nitrogen supply**	kno3	Constant for extractable soil nitrogen	0.02	-
nitrogen	nitrogen supply (potential)	total__n__uptake__max	Maximum total nitrogen uptake in a soil layer	0.6	g m-2
nitrogen	phenological stages shift (nitrogen)	N__fact__pheno	Multipler for N deficit effect on phenology	100	-
nitrogen	**leaf expansion (nitrogen)**	N__fact__expansion	Multipler for N deficit effect on leaf expansion	1	-
nitrogen	**photosynthesis (nitrogen)**	N__fact__photo	Multiplier for nitrogen deficit effect on photosynthesis	1.5	-
nitrogen	**leaf nitrogen remobilisation**	leaf__n__sen__conc	N concentration that remains in senesced leaves (i.e. that is not retranslocated)	0.005	g g-1
nitrogen	**grain filling (nitrogen)**	N__fact__grain	Multipler for N deficit effect on grain filling	1.5	-
nitrogen	**leaf nitrogen demand (min, crit, max)**	n__conc__leaf	Parameter added to change minimum, critical and maximum values of n__conc__leaf all at once	1	-
nitrogen	leaf nitrogen demand*	y__n__conc__crit__leaf	Critical leaf N concentration at the different stages	0.063 0.063 0.042 0.015 0.008 0.0035	g g-1
nitrogen	leaf nitrogen demand*	y__n__conc__max__leaf	Maximum leaf N concentration at the different stages	0.07 0.07 0.05 0.02 0.01 0.005	g g-1
nitrogen	leaf nitrogen demand*	y__n__conc__min__leaf	Minimum leaf N concentration at the different stages	0.025 0.025 0.021 0.01 0.005 0.0025	g g-1
nitrogen	**root nitrogen demand (min, crit, max)**	n__conc__root	Parameter added to change minimum, critical and maximum values of n__conc__root all at once	1	-
nitrogen	root nitrogen demand*	n__conc__crit__root	Critical root N concentration at the different stages	0.02	g g-1
nitrogen	root nitrogen demand*	n__conc__max__root	Maximum root N concentration at the different stages	0.02	g g-1
nitrogen	root nitrogen demand*	n__conc__min__root	Minimum root N concentration at the different stages	0.01	g g-1
nitrogen	root nitrogen remobilisation	root__n__sen__conc	N concentration that remains in the senesced roots (i.e. that is not retranslocated)	0.005	g g-1
nitrogen	**stem nitrogen demand (min, crit, max)**	n__conc__stem	Parameter added to change minimum, critical and maximum values of n__conc__stem all at once	1	-
nitrogen	stem nitrogen demand*	y__n__conc__crit__stem	Critical stem N concentration at the different stages	0.05 0.05 0.02 0.01 0.005 0.0035	g g-1
nitrogen	stem nitrogen demand*	y__n__conc__max__stem	Maximum stem N concentration at the different stages	0.07 0.07 0.04 0.015 0.015 0.015	g g-1
nitrogen	stem nitrogen demand*	y__n__conc__min__stem	Minimum stem N concentration at the different stages	0.025 0.025 0.004 0.003 0.003 0.0025	g g-1
nitrogen	stem nitrogen remobilisation	stem__n__sen__conc	N concentration that remains in the senesced stems (i.e. that is not retranslocated)	0.0025	g g-1

Module	Process	Factor	Description	Default Value	Unit
nitrogen	pod nitrogen demand (min, crit, max)	n_conc_pod	Parameter added to change minimum, critical and maximum values of n_conc_pod all at once	1	-
nitrogen	pod nitrogen demand*	y_n_conc_crit_pod	Critical rachis N concentration at the different stages	0.05 0.05 0.02 0.01 0.005 0.0035	g g-1
nitrogen	pod nitrogen demand*	y_n_conc_max_pod	Maximum rachis N concentration at the different stages	0.07 0.07 0.04 0.015 0.015 0.015	g g-1
nitrogen	pod nitrogen demand*	y_n_conc_min_pod	Minimum rachis N concentration at the different stages	0.025 0.025 0.004 0.003 0.003 0.0025	g g-1
nitrogen	pod nitrogen remobilisation	pod_n_sen_conc	N concentration that remains in the senesced rachis (spike without grains)	0.007	g g-1
nitrogen	meal nitrogen demand (min, crit, max)	n_conc_meal	Parameter added to change minimum, critical and maximum values of n_conc_meal all at once	1	-
nitrogen	meal nitrogen demand*	y_n_conc_crit_meal	Critical meal N concentration at the different stages	0 0 0 0 0.03 0.03	g g-1
nitrogen	meal nitrogen demand*	y_n_conc_max_meal	Maximum meal N concentration at the different stages	0 0 0 0 0.03 0.03	g g-1
nitrogen	meal nitrogen demand*	y_n_conc_min_meal	Minimum meal N concentration at the different stages	0 0 0 0 0.014 0.014	g g-1
nitrogen	meal nitrogen remobilisation	meal_n_sen_conc	N concentration that remains in senesced grains (i.e. that is not retranslocated)	0.014	g g-1
nitrogen	gain nitrogen demand (min, crit, max)	n_conc_grain	Parameter added to change minimum, critical and maximum values of n_conc_grain all at once	1	-
nitrogen	grain nitrogen demand*	n_conc_crit_grain	Critical grain N concentration at the different stages	0.03	g g-1
nitrogen	grain nitrogen demand*	n_conc_max_grain	Maximum grain N concentration at the different stages	0.03	g g-1
nitrogen	grain nitrogen demand*	n_conc_min_grain	Minimum grain N concentration at the different stages	0.014	g g-1
senescence	**leaf senescence (age)**	fr_lf_sen_rate	Fraction of total leaf number senescing per main stem node	0.035	[0-1]
senescence	**leaf senescence (age)**	node_sen_rate	Rate of node senescence on main stem	60	°Cd node-1
senescence	**leaf senescence (temperature)**	x_maxt_senescence	Minimum temperature affecting the daily fraction of leaf area senescing due to heat (y_heatsenescence_fac = 0 0.05 0.40)	34 34.1 45	°C
senescence	**leaf senescence (water)**	sen_rate_water	Slope in linear equation relating soil water stress during photosynthesis to leaf senescence rate	0.1	-
senescence	leaf senescence (carbon)	min_tpla	Lower limit of total leaf area per plant	5	mm2 plant-1
senescence	leaf senescence (shading)	lai_sen_light	Critical LAI when shading is starting to cause leaf area senescence	7	m2 m-2
senescence	leaf senescence (shading)	sen_light_slope	Sensitivity of leaf area senescence to shading	0.002	-
senescence	root senescence (nitrogen)	y_dm_sen_frac_root	Rate of root biomass senescing each day as a function of x_dm_sen_frac_root = 0 1	0.005 0.005	[0-1]

Table S1. Description of the APSIM-Wheat parameters included in the sensitivity analysis. *Module* refers to the sub-model where the parameter is used in APSIM-Wheat, *Process* refers to the physiological process targeted by the considered parameter and *Factor* is the parameter name used in the present study and in the APSIM-Wheat documentation (Zheng et al., 2014), where a complete description of the parameters is given. The *Default Value* field lists the nominal value of the parameter for cultivar *Hartog* in APSIM-Wheat 7.5 (only first three values were presented when the parameter is defined as a vector). In the *Process* field, influential parameters in indicated in bold and parameters that were grouped together for physiologic reasons are identified by (*).
